# Polarization insensitive symmetrical structured double negative (DNG) metamaterial absorber for Ku-band sensing applications

**DOI:** 10.1038/s41598-021-04236-1

**Published:** 2022-01-10

**Authors:** Mohammad Lutful Hakim, Touhidul Alam, Mohamed S. Soliman, Norsuzlin Mohd Sahar, Mohd Hafiz Baharuddin, Sami H. A. Almalki, Mohammad Tariqul Islam

**Affiliations:** 1 Pusat Sains Angkasa, Institut Perubahan Iklim, Universiti Kebangsaan Malaysia (UKM), 43600 Bangi, Selangor Malaysia; 2 Department of Electrical Engineering, College of Engineering, Taif University, P.O. Box 11099, Taif, 21944 Saudi Arabia; 3 Department of Electrical Engineering, Faculty of Energy Engineering, Aswan University, Aswan, 81528 Egypt; 4 Department of Electrical, Electronic and Systems Engineering, Faculty of Engineering and Built Environment, Universiti Kebangsaan Malaysia, 43600 Bangi, Malaysia

**Keywords:** Electrical and electronic engineering, Metamaterials

## Abstract

Metamaterial absorber (MMA) is now attracting significant interest due to its attractive applications, such as thermal detection, sound absorption, detection for explosive, military radar, wavelength detector, underwater sound absorption, and various sensor applications that are the vital part of the internet of things. This article proposes a modified square split ring resonator MMA for Ku-band sensing application, where the metamaterial structure is designed on FR-4 substrate material with a dielectric constant of 4.3 and loss tangent of 0.025. Perfect absorption is realized at 14.62 GHz and 16.30 GHz frequency bands, where peak absorption is about 99.99% for both frequency bands. The proposed structure shows 70% of the average absorption bandwidth of 420 MHz (14.42–14.84 GHz) and 480 MHz (16.06–16.54 GHz). The metamaterial property of the proposed structure is investigated for transverse electromagnetic mode (TEM) and achieved negative permittivity, permeability, and refractive index property for each absorption frequency band at 0°, 45°, and 90° polarization angles. Interference theory is also investigated to verify the absorption properties. Moreover, the permittivity sensor application is investigated to verify the sensor performance of the proposed structure. Finally, a comparison with recent works is performed, which shows that the proposed MMA can be a good candidate for Ku-band perfect absorber and sensing applications.

## Introduction

Veselago first theoretically introduced metamaterial double negative property (DNG) [negative permittivity and permeability] in 1968^1,2^. It was clarified in 2000 by Smith^2^, who proposed non-natural composite material with simultaneously negative permittivity and permeability. This feature results in a negative refractive index^3^. DNG property of metamaterial depends on its structure, which empowers it for different use like absorbers^4,5^, antennas, SAR (specific absorption ratio)^6^, filters^7^, waveguides^8^, invisible clocks^6^, mobile applications^9^, polarization converters^10^, lenses^11^, THz optical applications^12–14^ and different types of sensor^5,15–17^. After the first successful ascertainment of metamaterial absorber (MMA) properties, numerous studies are ongoing to design MMA for several applications like GPS positioning application^18^, noise reduction by improving MIMO antenna isolation^19^, reduction of radar cross-section^20^, stealth technology for military use^21^, underwater sound absorption^22^, cryptography^23^, thermal and wavelength selectable microbolometers^24^, cancer cell detection or biosensing^16,25,26^, imaging^27,28^ and detection for explosive materials^29^. Usually, a three-layer model is used to design MMA. The conductive material patch and ground plane are placed on the opposite side of the dialectic substrate, and the substrate generates coupling capacitance between ground and patch. Double-layer^30^ and multilayer^5,22^ models are also used for MMA design. To achieve a good encirclement of EM wave, the symmetrical structure is used for high absorption and distribution of surface current^31^. Usually, copper is used to design patches and ground, but the key concern is choosing the appropriate substrate. Although research is ongoing on developing substrate material due to the uniqueness of new substrate, commercial production and implementation in electromagnetic (EM) waves is a major task. Different substrates like Rogers RO or RT and FR-4 are commercially available and used recently due to their usability and availability^32^.

In recent times, several MMA structures have been developed for C, X and Ku-band applications. In^4^, a square spiral shape antenna is presented for the Ku-band absorption application, where 50° incident angle stability is achieved. In^33^, a four-square SRR (split-ring resonator)-based MMA has been developed for multi-band (C, X, Ku) absorption with 60° polarization angle stability and 99.38% of maximum absorption. A quarter SR with an inner asterisk resonator (AR) is presented in^34^, where polarization angle insensitivity and absorption increase. In^35^, three different absorbers for single (T shape), dual (split-I shape), and multi-band (Split Jerusalem Cross) applications are presented. A shorted stub circular ring MMA was proposed in^36^ for K-band application where 99.9% and 99.83% absorption with 70° polarization stability is achieved at 17 GHz and 18 GHz, respectively. In paper^37^, a cross-shaped resonator (CSR) and complementary cross-shaped resonator (CCSR) is presented for X and Ku frequency band applications with maximum absorption of 99.9% and incident angle stability of 60°. A polarization angle-sensitive circular ring resonator (CRR) is presented in^38^, where 99.66% peak absorption is realized at both 8.12 GHz and 12.39 GHz resonant frequencies. In^39^, a Jerusalem cross with a meandered load absorber is presented where 95% of average absorption is realized for three resonant frequency bands (14.75 GHz, 15.1 GHz, and 16.25 GHz). An eight-resistive arm (ERA) cell absorber MMA with 99% absorption and 65° angular stabilities is presented for X and Ku-band application^40^. To improve the absorption, an SRR structure is incorporated with a Jerusalem cross in ^41^. Moreover, research has been performed to enhance angular stability up to 75° in ^29^. A concentric closed CRR has been used to improve the angular stability of the absorber for C, X, and Ku-band applications. Besides, a modified CSRR is proposed for 90° angular insensitivity applications at X, Ku, and K bands^32^. A C-shaped MMA is presented for absorption applications in^15^, and a single resonating band is achieved in the Ku-band spectrum. In^42^, a compact polarization-insensitive MMA is proposed for C and X band absorbers. From the study, the perfect metamaterial absorber for a dedicated Ku band application that will not absorb other frequency band spectrum is rear. Moreover, most of them have larger unit cell size, single negative (SNG) [negative permittivity or permeability] metamaterial property and suffer from lower polarization insensitivity, which are the major limitations of MMA.

Based on previous research, this research proposed a compact modified SSRR structure with a cross strip line for dual-band absorption and sensing application at the Ku-band spectrum. The developed MMA structure apprehends 99.99% of absorption in Ku-band (14.62 GHz and 16.30 GHz) with broad incident angle stability. DNG property is also achieved for TE, TM, and TEM modes. Moreover, interference theory and PCR property are investigated to ensure the perfect absorber properties rather than polarization converter.

## Unit cell design and simulation setup

**Figure 1:**
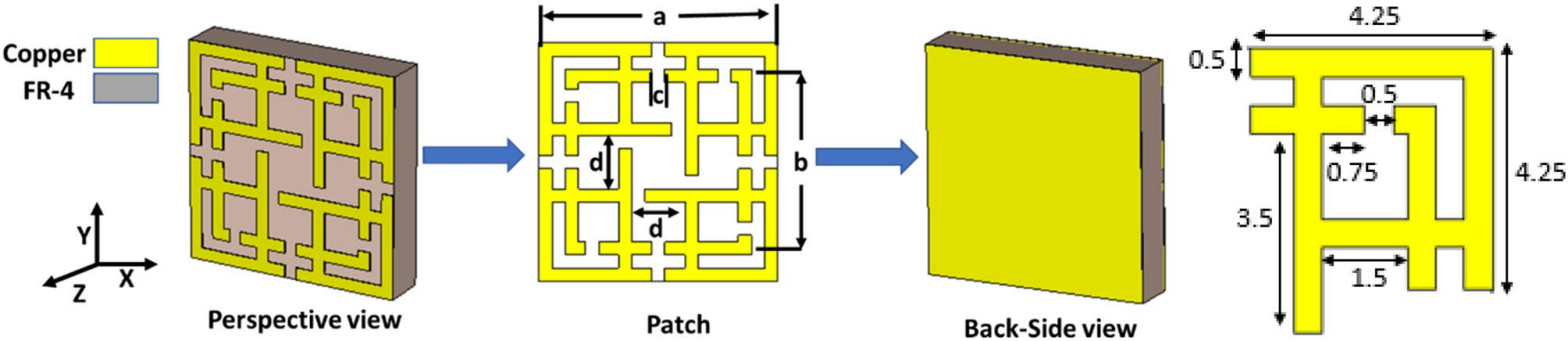
The proposed MMA unit cell with dimension (CST STUDIO SUITE 2019, https://www.3ds.com/products-services/simulia/products/cst-studio-suite)^45^.

The three-layer sandwich model (patch-substrate-ground) is a popular model for MMA design^15,29,31–39,41,42^. The proposed design structure uses this three-layer model. The structural dimension and resonator of MMA have a vital role in accomplishing absorption near unity. Different resonator structures like CSRR^31,36–38^, square spiral shape^4^, SSRR^32^, quarter SR with inner AR^33^, Jerusalem Cross^35,39^, eight-resistive arm cell^40^, and Ring C-shape quasi-MMA^15^ have been designed. Still, most of them suffer from limitations such as larger unit cell size, lower absorption and polarization insensitivity. The proposed Ku-band MMA has been designed for perfect absorption peak, polarization insensitivity and DNG property. The symmetrical MMA unit cell of the SSRR with microstrip line combination is shown in Fig. 1, where the copper patch is printed on a 1.6 mm thick FR-4 substrate with a dielectric constant of 4.3 and a loss tangent of 0.025. The metallic copper has a good conductivity, which creates inductance and capacitance to generate resonance at desired frequency. Low cost, durability, water resistivity and excellent insulation property between copper layers are the main advantages of using FR4 substrate material in the proposed MMA. The overall size of the MMA is 9 × 9 mm^2^. The proposed MMA unit cell consists of two SSRRs, where the length of outer and inner SSRR is a = 9.0 mm and b = 7.0 mm, respectively. The width between the splits and strapline is c = 0.5 mm, and microstrip line separation is d = 2.0 mm. Based on Wood's anomaly of electromagnetic (EM) wave absorption by the plane surface, the size of the unit cell was settled^43^. Inductive copper strips act as transmission lines for surface current to flow.

On the other hand, the gaps work as capacitive loads, which keep the electric dipole immersed and spread it among the copper strip on the substrate (FR-4) materials. The XY and the rotational symmetrical patch is designed in such a way that it will absorb all the entire EM waves at different frequencies. Proper boundary condition configuration is very important for effective MMA design and simulation in transverse electric (TE), transverse magnetic (TM), and transverse electromagnetic (TEM) modes^40,44^. The unit cell boundary condition has been applied along with the x-axis and y-axis for TE and TM mode. On the other hand, periodic boundary conditions are applied for TEM mode. EM wave applied along the positive z-axis. The proposed design has been simulated in computer simulation technology microwave studio (CST MWS). To attain accurate values, a frequency-domain solver has been used. Evaluation of the patch and parametric analysis for absorption property is discussed in the design analysis section.

## Design analysis

The evolution and corresponding metamaterial performances of the proposed MMA are described in this section. Based on Wood's anomaly for plane frequency selective surface (FSS)^43^, the SSRR structure has been modified to improve absorption value and DNG property. The absorption [$A(\omega )$] of the proposed MMA is calculated by Eq. (1)^46^, where $S_{11}$ and $S_{21}$ is the reflection coefficient and the transmission coefficient, respectively.

1 $$A(\omega ) = 1 - \left| {S_{11} } \right|^{2} - \left| {S_{21} } \right|^{2}$$

In the proposed design, copper ground plane use with permeability $\mu = 1$, resistivity $\rho = 1.72\Omega$, conductivity $\sigma = 5.8 \times 10^{7} \,{\text{S/m}}$ and the skin depth of EM wave is $\delta = \sqrt {{\rho \mathord{\left/ {\vphantom {\rho {\pi f\mu }}} \right. \kern-\nulldelimiterspace} {\pi f\mu }}}$ = 0.0062 mm. the copper ground thickness is 0.035 mm, which can block transmission of incident EM wave. So, Eq. (1) becomes,

2 $$A\left( \omega \right) = 1 - \left| {S_{11} } \right|^{2}$$

The reflection coefficient $\left( {S_{11} } \right)$ is significantly controlled by the effective impedance $\left( {Z_{eff} = \sqrt {{{\mu_{o} \mu_{r\left( \omega \right)} } \mathord{\left/ {\vphantom {{\mu_{o} \mu_{r\left( \omega \right)} } {\varepsilon_{o} \varepsilon_{r\left( \omega \right)} }}} \right. \kern-\nulldelimiterspace} {\varepsilon_{o} \varepsilon_{r\left( \omega \right)} }}} } \right)$ of the MMA, which can be understood from Eq. (3)^47^,

3 $$S_{11} = \frac{{Z_{eff} - Z_{o} }}{{Z_{eff} + Z_{o} }}$$

where free space impedance $Z_{o} = \sqrt {{{\mu_{o} } \mathord{\left/ {\vphantom {{\mu_{o} } {\varepsilon_{o} }}} \right. \kern-\nulldelimiterspace} {\varepsilon_{o} }}} \approx 377\Omega$. $\mu_{r\left( \omega \right)}$ and $\varepsilon_{r\left( \omega \right)}$ is the frequency dependent permeability and permittivity respectively and $\mu_{o}$ and $\varepsilon_{o}$ is the free space permeability and permittivity consequently. By equating Eqs. (2) and (3) absorption equation stands,

4 $$A\left( \omega \right) = \frac{{2Z_{o} }}{{{\text{Re}} \left| {Z_{eff} } \right| + i\cdot{\text{Im}} \left| {Z_{eff} } \right| - Z_{o} }}$$

The maximum absorption A = 1 achieved for the condition of ${\text{Re}} \left| {Z_{eff} } \right| \approx 377\Omega$ and ${\text{Im}} \left| {Z_{eff} } \right| \approx 0$. The reflection happens if the incident EM wave undergoes a mismatch of free space impedance $Z_{o} = 377\Omega + j0$. The absorption property sensitively depends on the complex value of permeability and permittivity when the impedance matching condition happens, and there is no wave reflection. The normalized impedance is calculated by Eq. (5)^35^.

5 $$Z = {{Z_{eff} } \mathord{\left/ {\vphantom {{Z_{eff} } {Z_{o} = \sqrt {{{\mu_{r} } \mathord{\left/ {\vphantom {{\mu_{r} } \varepsilon }} \right. \kern-\nulldelimiterspace} \varepsilon }_{r} } }}} \right. \kern-\nulldelimiterspace} {Z_{o} = \sqrt {{{\mu_{r} } \mathord{\left/ {\vphantom {{\mu_{r} } \varepsilon }} \right. \kern-\nulldelimiterspace} \varepsilon }_{r} } }} = \sqrt {{{\left( {1 + S_{11} } \right)^{2} - S_{21}^{2} } \mathord{\left/ {\vphantom {{\left( {1 + S_{11} } \right)^{2} - S_{21}^{2} } {\left( {1 - S_{11} } \right)^{2} - S_{21}^{2} }}} \right. \kern-\nulldelimiterspace} {\left( {1 - S_{11} } \right)^{2} - S_{21}^{2} }}}$$

**Figure 2:**
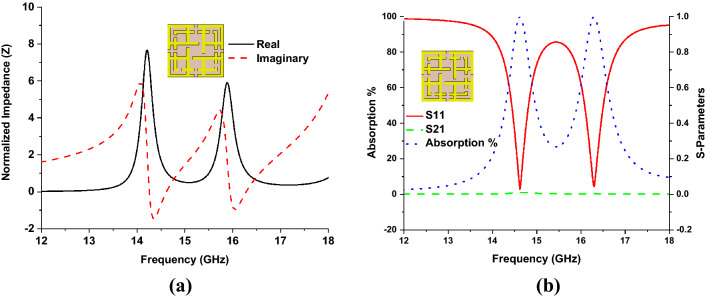
(**a**) Normalized impedance and (**b**) S-parameters and absorption of the designed MMA.

By equating Eqs. (4) and (5), the relation between $\eta$ and $Z$ can be derived as $Z = {\eta \mathord{\left/ {\vphantom {\eta {\varepsilon_{r} }}} \right. \kern-\nulldelimiterspace} {\varepsilon_{r} }}$. So complex value of $\eta$, both real and imaginary part sensitivity plays a vital role in impedance matching. Figure 2a shows the real and imaginary part of the normalized impedance is nearer to unity and zero, which achieved unity absorption at 14.62 GHz and 16.30 GHz frequency. The s-parameters and absorption plots for the proposed MMA are shown in Fig. 2b.

**Figure 3:**
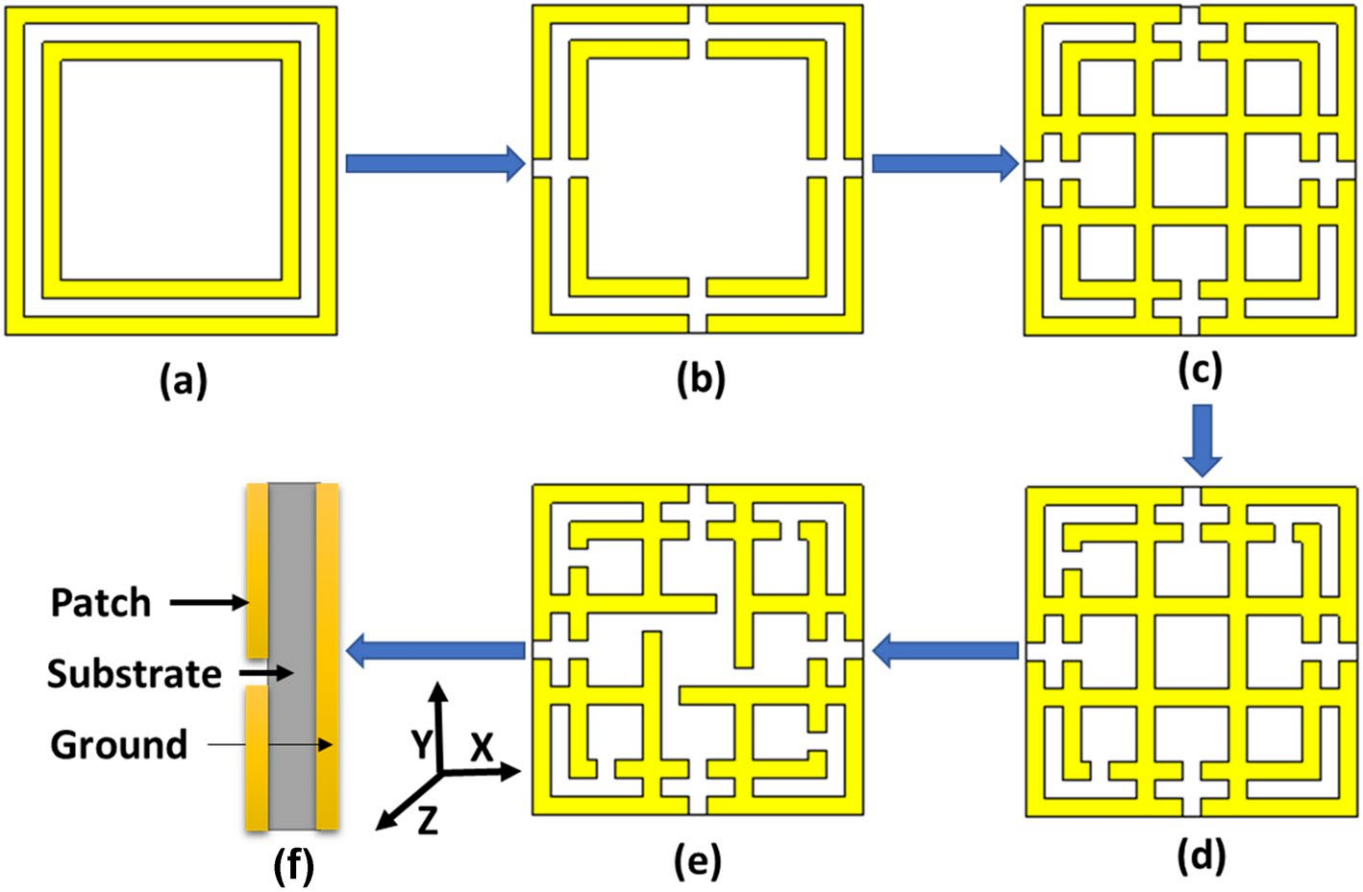
Evaluation of proposed unit cell (**a**) unit cell with only square ring resonator (**b**) unit cell with square split ring resonator (SSRR) (**c**) SSRR with microstrip line two in the x-axis and another two in the y-axis (**d**) making another four splits in inner SRR (**e**) final design (**f**) side view of the final design (CST STUDIO SUITE 2019, https://www.3ds.com/products-services/simulia/products/cst-studio-suite)^45^.

**Figure 4:**
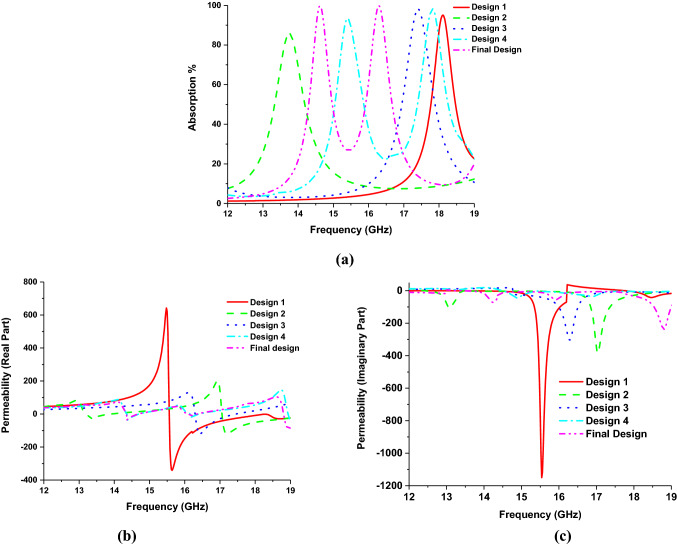

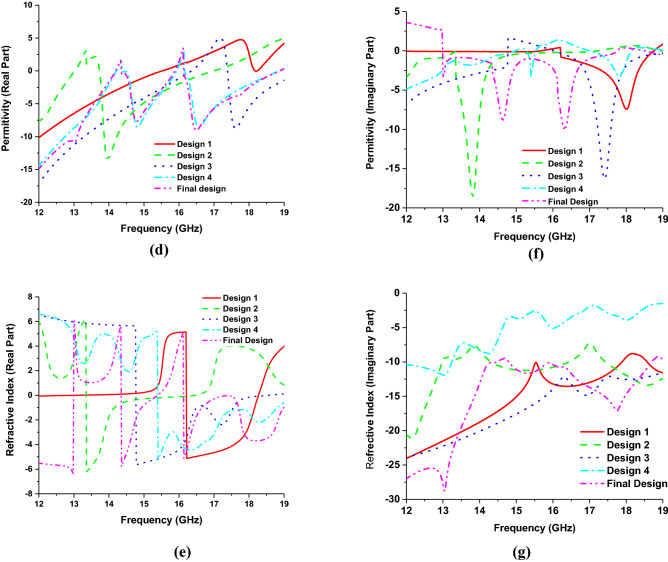
Metamaterial property of different design models. (**a**) Absorption characteristics, (**b**) permeability (real), (**c**) permeability (imaginary), (**d**) Permittivity (real), (**e**) permittivity (imaginary) (**f**) refractive Index (real) and (**g**) refractive index (imaginary).

The metamaterial properties of the proposed MMA structure are calculated from the complex value of s-parameters (scattering parameters), which are derived by the Nicolson–Ross–Weir (NRW) formula discussed in^46^.

6 $$\varepsilon_{r} = {\raise0.7ex\hbox{$2$} \!\mathord{\left/ {\vphantom {2 {jk_{0} d}}}\right.\kern-\nulldelimiterspace} \!\lower0.7ex\hbox{${jk_{0} d}$}} \times {\raise0.7ex\hbox{${\left( {1 - S_{11} - S_{21} } \right)}$} \!\mathord{\left/ {\vphantom {{\left( {1 - S_{11} - S_{21} } \right)} {\left( {1 + S_{11} + S_{21} } \right)}}}\right.\kern-\nulldelimiterspace} \!\lower0.7ex\hbox{${\left( {1 + S_{11} + S_{21} } \right)}$}}$$

7 $$\mu_{r} = {\raise0.7ex\hbox{$2$} \!\mathord{\left/ {\vphantom {2 {jk_{0} d}}}\right.\kern-\nulldelimiterspace} \!\lower0.7ex\hbox{${jk_{0} d}$}} \times {\raise0.7ex\hbox{${\left( {1 - S_{21} + S_{11} } \right)}$} \!\mathord{\left/ {\vphantom {{\left( {1 - S_{21} + S_{11} } \right)} {\left( {1 + S_{21} - S_{11} } \right)}}}\right.\kern-\nulldelimiterspace} \!\lower0.7ex\hbox{${\left( {1 + S_{21} - S_{11} } \right)}$}}$$

8 $$\eta = \sqrt {\varepsilon_{r} \mu_{r} }$$

where $\varepsilon_{r}$ = permittivity, $\mu_{r}$ = permeability, $\eta$ = refractive index, wave number $k_{0} = {{2\pi f} \mathord{\left/ {\vphantom {{2\pi f} c}} \right. \kern-\nulldelimiterspace} c}$, where $f$ is the microwave frequency and $c$ is the velocity of light, $d$ = substrate thickness. Since the values of $k_{0}$ and $d$ are constant, on the other hand, the transmission coefficient ($S_{21}$) is zero; hence the equation of permittivity becomes $\varepsilon_{r} = {2 \mathord{\left/ {\vphantom {2 {jk_{0} d}}} \right. \kern-\nulldelimiterspace} {jk_{0} d}} \times {{(1 - S_{11} )} \mathord{\left/ {\vphantom {{(1 - S_{11} )} {(1 + S_{11} )}}} \right. \kern-\nulldelimiterspace} {(1 + S_{11} )}}$. All the calculations are done by MATLAB software. The reflection coefficient value ($S_{11}$) significantly contributes to the negative values of permittivity and permeability, which is determined by the design evaluation of the MMA patch shown in Fig. 3. In the first design step, two square rings have been placed on a substrate material to form dual absorption peaks with a single negative (SNG) property and negative refractive index. To achieve double negative (DNG) properties, four splits are made in each ring on each side to create coupling capacitance, but the absorption is decreased. Two microstrip lines are placed at *d* distance from each other along the x-axis. Another two are placed at the same distance along the y-axis, which divides the unit cell into the different segments and increases the inductive effect to increase the absorption level for a single frequency with DNG properties. By doing four splits in the inner ring, double absorption peaks are achieved with an increased absorption level with DNG properties. Finally, by creating four splits in the microstrip line, dual-absorption peaks were achieved at 14.62 GHz and 16.30 GHz with unity absorption and large DNG bandwidth which makes the proposed one preferable over previous research^4,10,15,31,35–41^. The absorption, permeability (real), permeability (imaginary), permittivity (real), permittivity (imaginary), refractive index (real) and refractive index (imaginary) for the corresponding designs are shown in Fig. 4a–g, respectively.

**Figure 5:**
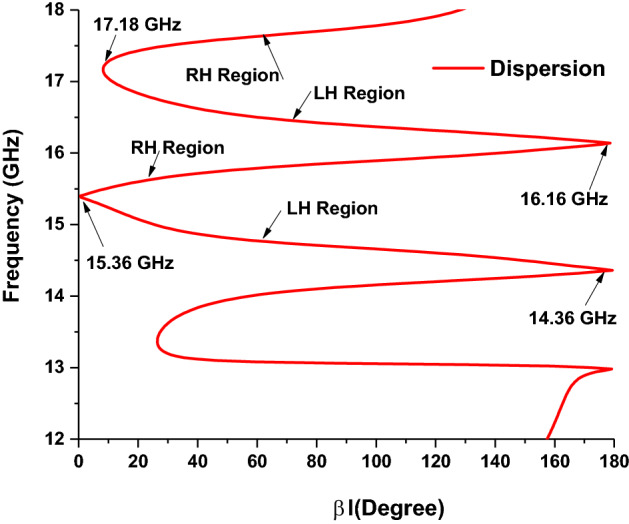
Dispersion diagram of the proposed MMA.

For a better understanding of metamaterial behaviours, the dispersion diagram of the proposed structure has been analyzed using Eq. (9)^42^, shown in Fig. 5. From Fig. 5, it is shown that both (14.42–14.84 GHz and 16.06–16.54 GHz) absorption frequency band lays in LH (left-handed) region slop is the negative value where permittivity, permeability and refractive index are also in the negative region. On the other side, in RH (right-handed) region, the slope is positive and permittivity, permeability refractive index is also positive, and, i.e. phase and group velocity are in parallel.

9 $$\beta l = \cos^{ - 1} \left( {\frac{{1 - S_{11} S_{22} + S_{12} S_{21} }}{{2S_{21} }}} \right)$$

**Figure 6:**
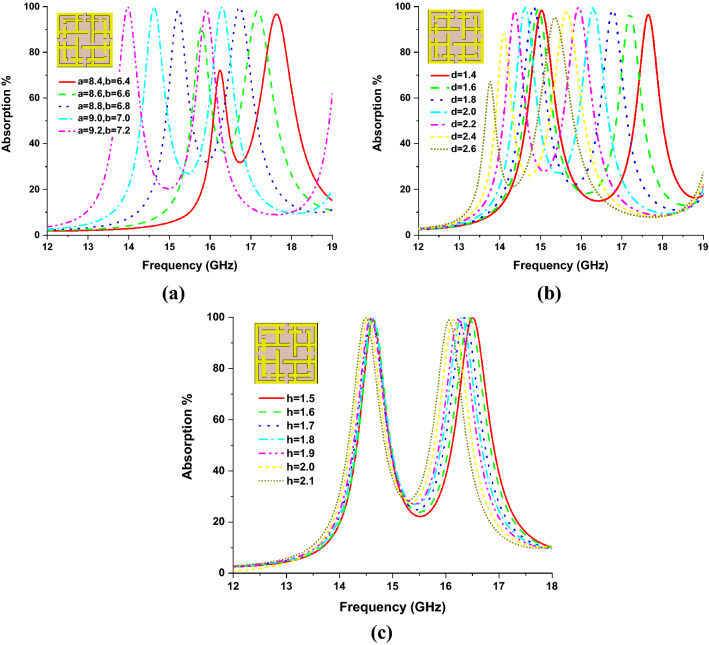
Absorption characteristics of parametric investigation (**a**) absorption of various lengths of inner and outer SSRR, (**b**) absorption of different values of microstrip line distance in x and y-axis and (**c**) absorption of different substrate thickness.

Parametric investigation of the proposed MMA is also presented for understanding the absorption behavior of the proposed MMA. By simultaneously increasing the SSRR length *a* and *b,* the absorption bandwidth can be shifted to the lower frequency band. Similarly, by decreasing the values, it can be shifted to the upper-frequency band. Figure 6a shows the absorption behavior of the proposed MMA unit cell for different lengths of the inner and outer SSRR structure. The absorption performances of the MMA are also investigated for different values of *d* parameter, which is the microstrip line distance in the x and y-axis. For *d* = 1.4 to 2.6 (0.2 mm apart), absorption characteristics are shown in Fig. 6b; the upper band shifts to upper frequency when d value decreases and shifts to lower frequency when d value increases. The effect of substrate thickness is shown in Fig. 6c, where it is observed that for different values of substrate thickness h, the lower absorption band is fixed in a position, where for the increment of h, the upper absorption band is slightly moved to the lower frequency band, which is only $\Delta f \approx 0.06$ MHz for the increment of h = 0.1 mm.

## Result analysis

### Interference theory

**Figure 7:**
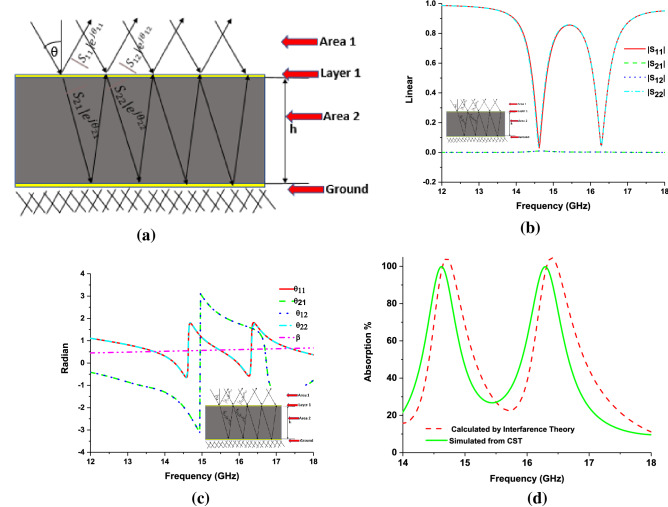
(**a**) Interference theory model, (**b**) s-parameter (linear), (**c**) phase, (**d**) absorption calculated from interference theory.

The absorption of the proposed MMA is also calculated by using interference theory based on^48–50^. From Fig. 7a, considering the thickness of layer 1 (copper) is zero and area 1 is air, area 2 is a dielectric substrate, the reflection coefficient of layer 1 (area 1 to 1) is $S_{11} = \left| {S_{11} } \right|e^{{j\theta_{11} }}$ the transmission coefficient of layer 1 (area 1 to 2) is $S_{21} = \left| {S_{21} } \right|e^{{j\theta_{21} }}$, the transmission coefficient of layer 1 (area 2 to 1) is $S_{12} = \left| {S_{12} } \right|e^{{j\theta_{12} }}$, the reflection coefficient of layer 1 (area 2 to 2) is $S_{22} = \left| {S_{22} } \right|e^{{j\theta_{22} }}$ EM wave applied in the positive z-direction, and the negative z-direction copper ground placed and also electric (Et = 0) applied, which work as a reflector. Figure 7b, c shows the linear value of s-parameters and phase value in radian. For layer 1, total $S_{11total}$ (reflection coefficient) is calculated by Eq. (10),

$$\begin{aligned} S_{11total} & = S_{11} + S_{12} e^{ - j\beta } e^{ - j\pi } e^{ - j\beta } S_{21} + S_{12} e^{ - j\beta } e^{ - j\pi } e^{ - j\beta } S_{21} (S_{22} e^{ - j\beta } e^{ - j\pi } e^{ - j\beta } )^{1} S_{21} \\ & \quad + S_{12} e^{ - j\beta } e^{ - j\pi } e^{ - j\beta } S_{21} (S_{22} e^{ - j\beta } e^{ - j\pi } e^{ - j\beta } )^{2} S_{21} + \cdots \cdots \\ \end{aligned}$$

$$S_{11total} = S_{11} + S_{12} e^{ - j(2\beta + \pi )} S_{21} \sum\limits_{n = 0}^{\alpha } {\left( {S_{22} e^{ - j(2\beta + \pi )} } \right)^{n} }$$

10 $$S_{11total} = \left| {S_{11} } \right|e^{{j\theta_{11} }} + {\raise0.7ex\hbox{${\left| {S_{12} } \right|\left| {S_{21} } \right|e^{{ - j( - \theta_{12} - \theta_{21} + 2\beta + \pi )}} }$} \!\mathord{\left/ {\vphantom {{\left| {S_{12} } \right|\left| {S_{21} } \right|e^{{ - j( - \theta_{12} - \theta_{21} + 2\beta + \pi )}} } {1 - \left| {S_{22} } \right|e^{{ - j( - \theta_{22} + 2\beta + \pi )}} }}}\right.\kern-\nulldelimiterspace} \!\lower0.7ex\hbox{${1 - \left| {S_{22} } \right|e^{{ - j( - \theta_{22} + 2\beta + \pi )}} }$}}$$

Proposed designed MMA is passive due to this $\left| {S_{12} } \right| \approx \left| {S_{21} } \right|$ so Eq. (6) becomes,

11 $$S_{11total} = \left| {S_{11} } \right|e^{{j\theta_{11} }} + {\raise0.7ex\hbox{${\left| {S_{12} } \right|^{2} e^{{j(2\theta_{12} - 2\beta - \pi )}} }$} \!\mathord{\left/ {\vphantom {{\left| {S_{12} } \right|^{2} e^{{j(2\theta_{12} - 2\beta - \pi )}} } {1 - \left| {S_{22} } \right|e^{{j(\theta_{22} - 2\beta - \pi )}} }}}\right.\kern-\nulldelimiterspace} \!\lower0.7ex\hbox{${1 - \left| {S_{22} } \right|e^{{j(\theta_{22} - 2\beta - \pi )}} }$}}$$

In Eq. (11), β = Kd = propagation constant phase, d is the transmitted wave propagation distance for layer 1 to the ground and k is the wavenumber. MATLAB software is used for calculating the Eq. (11). By using Eq. (2), we can calculate the absorption of proposed MMA where due to copper ground, transmission coefficient becomes zero and absorption depends only on scattering-parameter (reflection coefficient [S11total]) so absorption Eq. (1) becomes,

$$A(\omega ) = 1 - \left| {S_{11total} } \right|$$

Figure 7d shows the calculated absorption from interference theory, where the calculated absorption of interference theory is slightly distorted with Eq. (1) calculation due to the complex wavenumber of the dielectric substrate medium^51^.

### Equivalent circuit model (ECM) analysis

**Figure 8:**
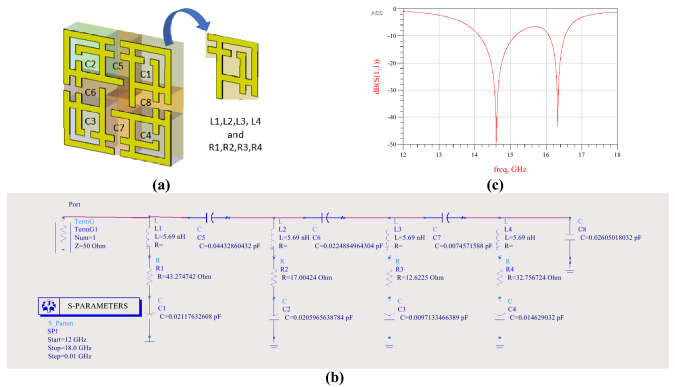
(**a**) The segment of the MMA patch, (**b**) equivalent circuit. Model (ECM), (**c**) S_11_ of the ECM (PathWave Advance Design System (ADS), https://www.keysight.com/sg/en/lib/resources/software-releases/pathwave-ads-2019.html)^52^.

Using advance design system (ADS) software, the lumped circuit of the proposed MMA has been illustrated in Fig. 8^5^. As designed, the MMA unit cell is shown in Fig. 1 divided into four segments, where each segment (shown in Fig. 8a) considers as an RLC circuit. The constant inductance L1, L2, L3, and L4 are calculated for four different segments by Eq. (12)^5^.

12 $$L_{s} = 0.00508L\left[ {\ln \left( {\frac{2L}{{W + D}}} \right) + 0.5 + 0.2235\frac{{\left( {W + D} \right)}}{L}} \right]$$

where *L* is the length of the stripline of the segment, *W* is the width of the strip, *D* is the substrate height (in inch), and Ls is the inductance in µH. The associated inner segment capacitances C1, C2, C3, C4 and coupling capacitance between the segments C5, C6, C7 and C8, are also considered. The capacitance (C1 and C3) is the most influential for desired frequency (*f*), which is calculated by using Eq. (13)^5^.

13 $$C = \frac{1}{{4\pi^{2} f^{2} L_{s} }}$$

The coupling capacitance is calculated by using Eq. (14), where *A* and *d* presents the area of the strip and the gap between the gap respectively. $\varepsilon_{o}$ and $\varepsilon_{r}$ is the relative permittivity of free space and medium respectively.

14 $$C = \varepsilon_{o} \varepsilon_{r} \frac{A}{d}$$

To find a similar reflection coefficient plot, the capacitance effect of each segment and associated resistance are tuned, where *C* is tuned for fixing resonant frequency, and R is tuned for increment or decrement of the reflection coefficient. Figure 8c shows the S_11_ plot from ADS.

### TE, TM and TEM mode absorption

**Figure 9:**
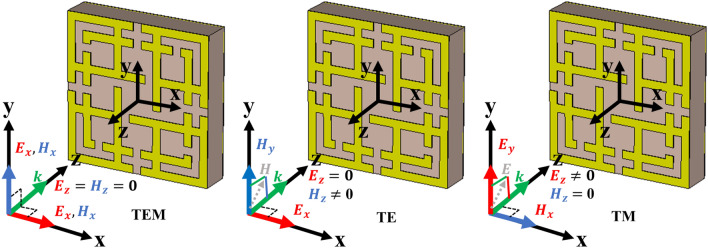
E-field, H-field and propagation direction for different mode (TEM, TE and TM). (CST STUDIO SUITE 2019, https://www.3ds.com/products-services/simulia/products/cst-studio-suite)^45^.

**Figure 10:**
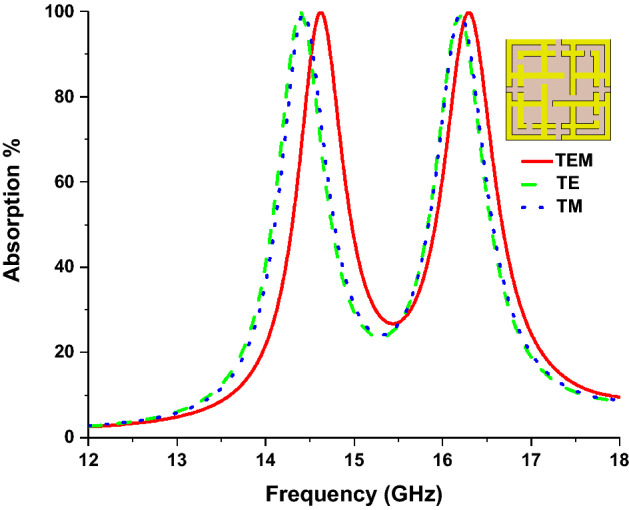
Absorption characteristics for TEM, TE, and TM mode.

Figure 9 shows the electric and magnetic field vectors for TEM, TE and TM mode. In TEM mode, simulation the direction of electric (E_x_) and magnetic (H_x_) field vectors both are transverse with wave propagation vector (k) along the z-axis, and there is no electric and magnetic field component towards wave propagation direction. In TE mode, only electric field vectors are transverse with k, where Magnetic field appears along propagation direction with zero electric fields. In TM mode, magnetic field vectors are transverse, and electric field vectors are not zero towards propagation direction z. Figure 10 shows the absorption of the MMA for transverse electromagnetic mode (TEM), transverse magnetic mode (TM), and transverse electric mode (TE). Due to the symmetrical design structure, near-unity absorption is achieved at 14.62 GHz and 16.30 GHz frequency for TEM modes, shown in Fig. 10. It can be noted that the lower band absorption peak for TE and TM mode slightly shifted to a lower frequency, although shifting in the higher frequency band is very negligible. For TEM mode, above 70% of average absorption has been realized for both frequency bands (14.42–14.84 GHz and 16.6–16.54 GHz). And for TE and TM mode, 70% of the average absorption is achieved for 14.22–15.06 GHz and 15.98–16.88 GHz, respectively. The energy storing capacity and sensitivity of the proposed MMA can also be understood from high-quality factor (Q) values of 34.80 and 33.95 at 14.62 GHz and 16.30 GHz frequency, respectively. This calculation is performed using $Q = {{f_{o} } \mathord{\left/ {\vphantom {{f_{o} } {HMBW}}} \right. \kern-\nulldelimiterspace} {HMBW}}$^53^, where *f*_*o*_ is the centre frequency and HMBW (half-power maximum bandwidth).

### PCR value analysis

**Figure 11:**
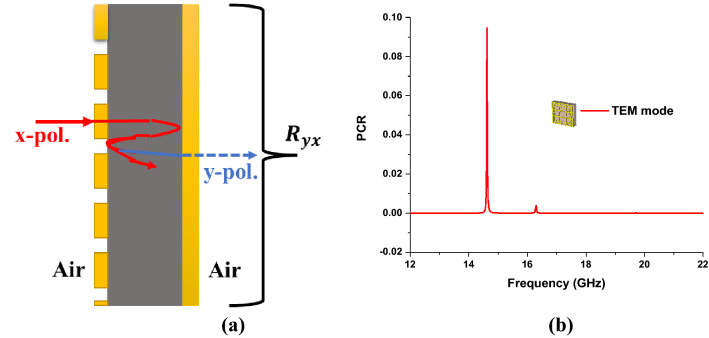
(**a**) x-polarization to y-polarization conversion (**b**) PCR value for TEM mode.

The Co & Cross Polarization and Polarization Conversion Ratio (PCR) value of the proposed MMA has been analyzed and presented in Fig. 11, calculated by Eq. (15). The cross-polarization of the proposed MMA unit cell is almost zero in the entire frequency range, which certifies that the unit cell cannot convert the x-polarized incident wave into the y-polarized reflected wave shown in Fig. 11.

15 $$PCR = \frac{{R_{yx}^{2} }}{{R_{yx}^{2} + R_{xx}^{2} }}$$

where $\left| {S_{11} (\omega )} \right|^{2} = R_{xx}^{2}$ = Co-polarized wave. Moreover, due to the copper back, the cross-polarization is zero, which is equal to the transmission coefficient, $\left| {S_{21} (\omega )} \right|^{2} = R_{yx}^{2}$ = cross-polarized wave. So, the PCR value for operating absorption bandwidth is almost zero for TEM mode, which is the ideal value of PCR for MMA^54^.

### TEM mode polarization angle analysis

**Figure 12:**
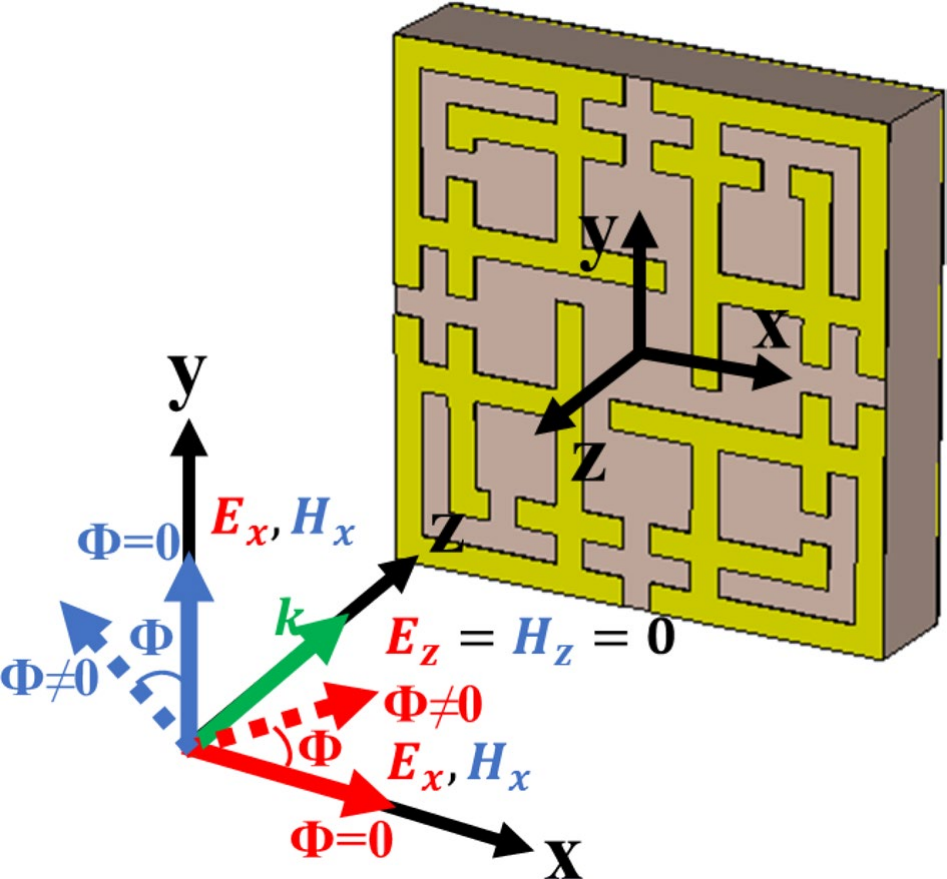
Direction of e-field and h-field for polarization angle zero and non-zero at TEM mode. (CST STUDIO SUITE 2019, https://www.3ds.com/products-services/simulia/products/cst-studio-suite)^45^.

**Figure 13:**
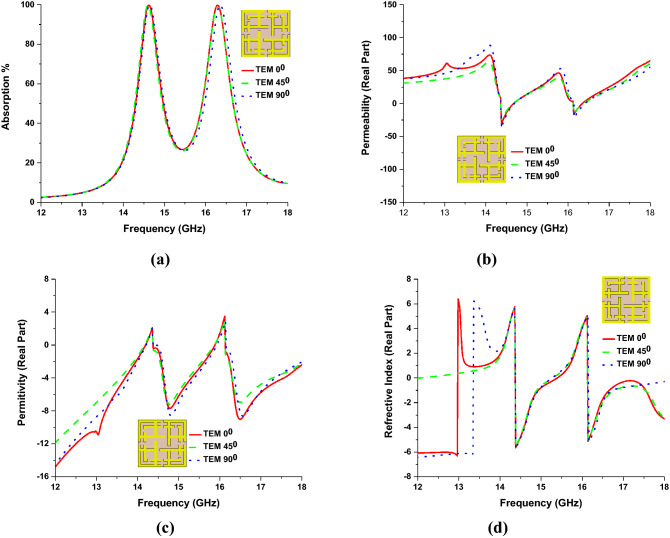
(**a**) absorption, (**b**) permeability [Real Part], (**c**) permittivity [Real Part], (**d**) Refractive Index [Real Part] curve for 0°, 45° and 90° polarization angles in TEM mode.

Figure 12 shows the direction of electric and magnetic field vectors of TEM propagation of the wave. For polarization angle zero (ф = 0), the direction of e-filed and h-field is along the x and y-axis of the MMA structure. This e and h field component is deflected with a certain angle for non-zero polarization angle. Simulated results of the proposed unit cell for TEM mode of incident EM wave (normal incident) are shown in Table 1, where simulation has been performed for different polarization angles. Two absorption peaks are found with similar bandwidth and DNG properties for each frequency, which is unique among relevant works. Maximum 99.99% of peak absorption is realized at Ku frequency bands (at 14.62 GHz and 16.30 GHz) with 420 MHz and 480 MHz bandwidth (− 10 dB or above 70%). In Table 1, the permittivity, permeability, and refractive index of the proposed MMA are presented. Figure 13a–d shows the absorption, permeability permittivity, and refractive index plot for different polarizing angles (0°, 45° and 90°) for TEM mode.

**Table 1:** Proposed unit cell performance for different polarization angles at TEM mode.

Polarizing angle	Frequency band	Resonant frequency (GHz)	Absorption bandwidth (GHz)	Permittivity	Permeability	Refractive index (NRW)	Maximum absorption (%)
0°	Ku	14.62	0.42	− 3.61	− 3.60	− 3.60	99.99
		16.30	0.48	− 4.13	− 2.94	− 3.48	99.99
45°	Ku	14.62	0.42	− 4.53	− 2.40	− 3.29	99.99
		16.30	0.48	− 3.90	− 0.39	− 3.63	99.99
90°	Ku	14.62	0.42	− 2.57	− 5.15	− 3.63	99.99
		16.30	0.48	− 1.92	− 7.44	− 3.77	99.99

### E-field, H-field and Surface Current Distribution

**Figure 14:**
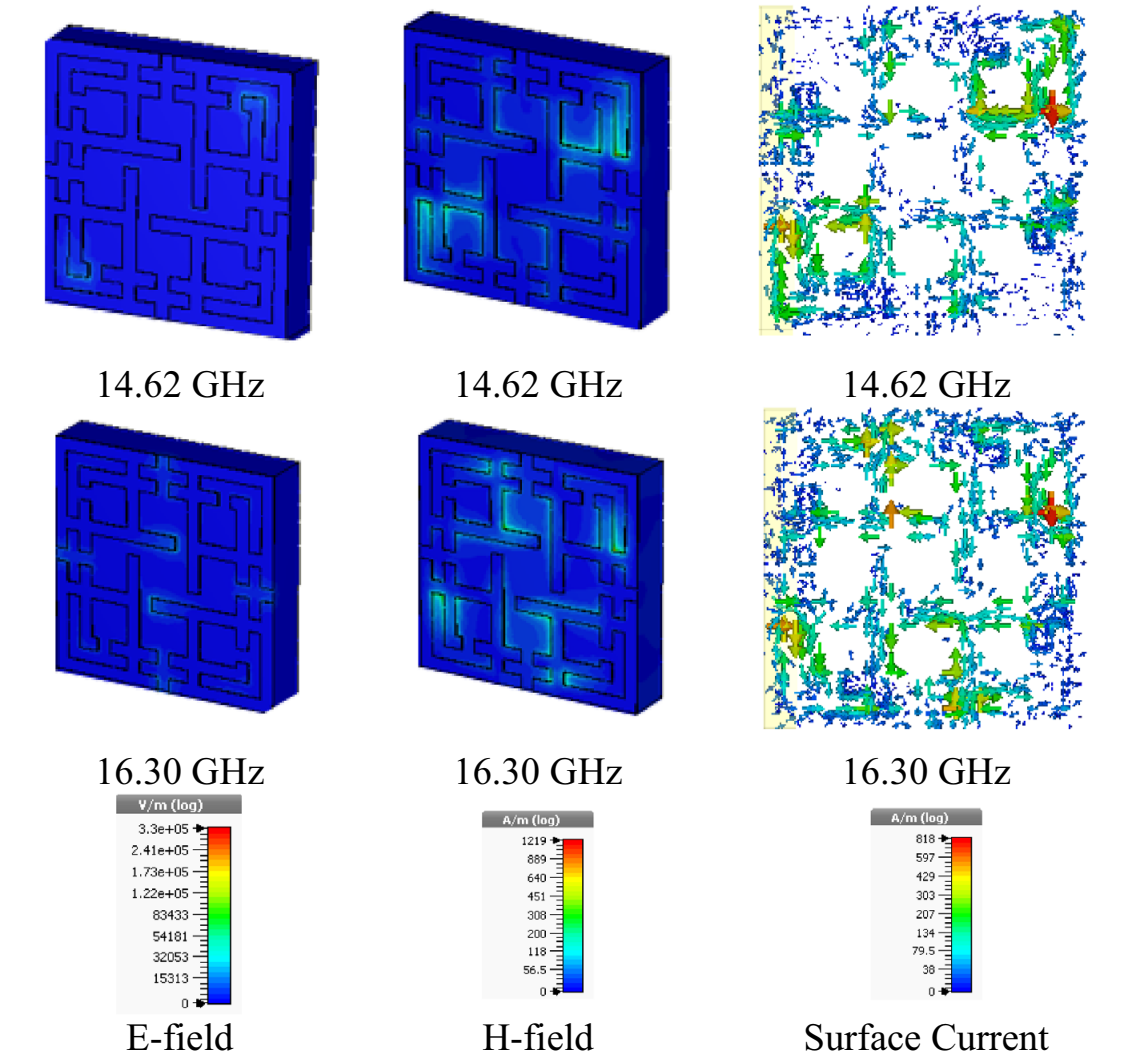
E-field, H-field, and surface current distribution of the proposed metamaterial absorber (CST STUDIO SUITE 2019, https://www.3ds.com/products-services/simulia/products/cst-studio-suite)^45^.

Figure 14 shows the e-field, h-field, and surface current distribution of the proposed MMA. By analyzing it, the behavior of the design structure can be adequately understood. From Fig. 14, it is observed that the h-field is much more agitated than the e-field. The red color indicates the higher density of e-field, h-field, and surface current. In E-field, at 14.62 GHz frequency, the upper-right and lower-left corner of the structure shows the higher field intensity. Besides, at 16.30 GHz frequency, an increased amount of field intensity shows in the middle of all four edges and the center of the structure. This occurs because of current flow through all the patches, and the direction of current flow changes with respect to frequency. Moreover, due to symmetrical structure, magnetic dipole associated with incident polarized h-field, where h-field is stuck and guided less reflection of EM wave and rampant absorption of structure.

### Experimental analysis

**Figure 15:**
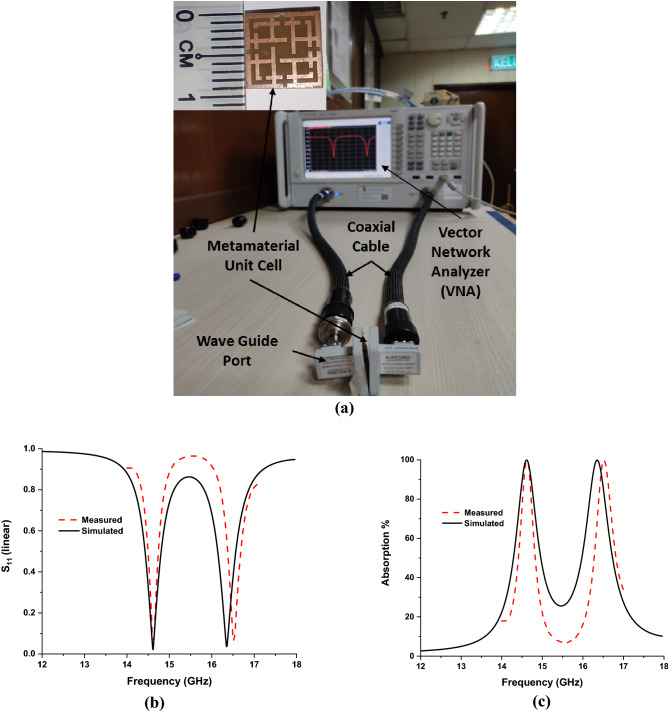
Measurement of proposed MMA (**a**) Setup, (**b**) S-parameters and (**c**) absorption.

The proposed MMA has been fabricated and measured to verify the electromagnetic properties of the proposed MMA, such as S11 and absorption property. Figure 15a shows the measurement setup, where the Vector Network Analyzer (VNA) and two waveguides (A-INFOMW, P/N:51WCAS_Cu) are used to measure S-parameters of the proposed MMA. Figure 15b, c shows S_11_ and absorption behavior of the developed MMA. The peak resonance in simulated and measured results are identical, though a little mismatch is observed at the upper frequency band. This discrepancy may be occurred due to fabrication tolerance, measurement tolerance and RF feeding cable, which were not possible to consider entirely in the simulation.

## Sensor applications

**Figure 16:**
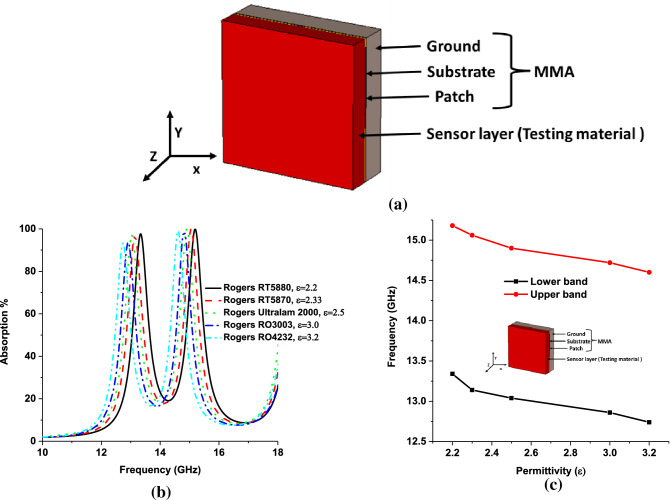
(**a**) Solid material sensing, (**b**) absorption characteristics of different testing materials and (**c**) peak absorption frequency plot of solid material sensing. (CST STUDIO SUITE 2019, https://www.3ds.com/products-services/simulia/products/cst-studio-suite)^45^.

**Figure 17:**
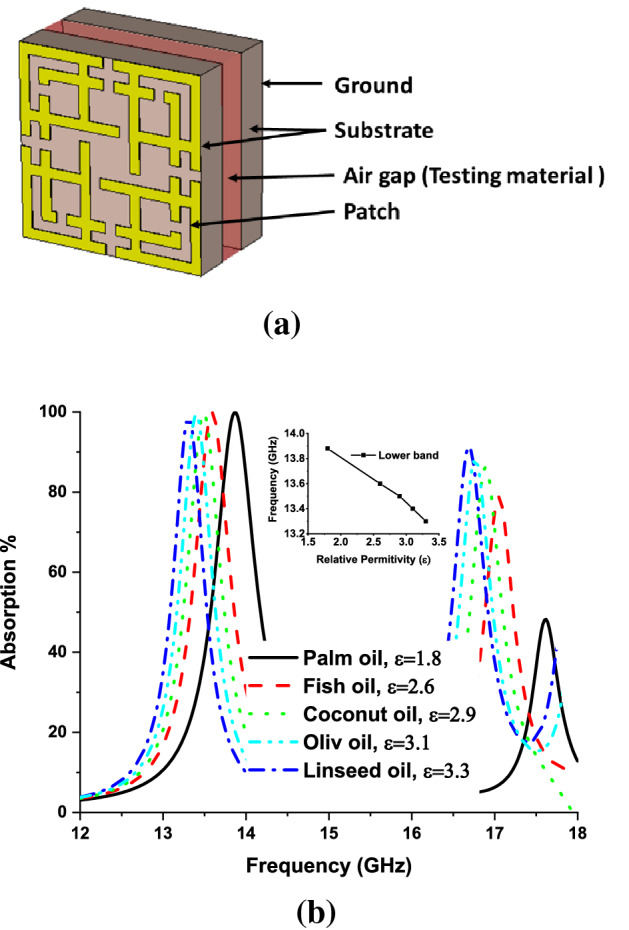
(**a**) Liquid material sensing, (**b**) absorption characteristics of water and glycerin. (CST STUDIO SUITE 2019, https://www.3ds.com/products-services/simulia/products/cst-studio-suite)^45.^

The outstanding performance of absorbing the EM signal of the MMA leads to its application in different aspects. Generally, MMA is designed for radio to THz frequency range like energy harvesting, THz application, undesired frequency absorption, optical switch, thermal emitter, and metamaterial sensor application^5^, various metamaterial sensors like permittivity sensor^55^, refractive index sensor^53^ base on absorption property, temperature sensor, chemical sensor, microfluid sensor, and pressure sensor^5,15,44,56–58^. The relation between permittivity and effective impedance can be understood from Eq. (5). The effective impedance plays a significance role in reflection and absorption of incident EM wave, which is discussed in Eqs. (3) and (4). The variation of permittivity leads to a consequence variation in the reflection and absorption^58^. Based on^15,44^, the permittivity sensor is investigated using the absorption properties of the proposed metamaterial. The sensor setup is illustrated in Fig. 14a, where for solid material sensing 1.5 mm thick sensor layer is placed on top of the MMA. Dielectric constant has a direct relation with permittivity. In normal state, the sensing layer is considered as air with dielectric constant 1. The dielectric constant of the solid sensing layer generates a significant variation in reflection and absorption of MMA by varying the effecting impedance of MMA. The variation of effective impedance significantly impacts the self-capacitance of the MMA patch, which leads to a shift in the absorption peak. Different testing materials (permittivity in the range of 2.2–3.2) are placed in the sensor layer to investigate the sensing performance. The investigated results are shown in Fig. 16b, c. For the increment of permittivity value, the frequency-shifted linearly from the higher to a lower frequency. In addition, a liquid material sensing investigation is also performed, illustrated in Fig. 17a. The air gap between the substrate material filled up by liquid material. The sensing performance has been investigated for different types of Edible oil (liquid). The absorption responses for different liquid are presented in Fig. 17b, where absorption peaks shifted to lower frequency with increasing permittivity values. The absorption of upper band is reduced due to changing in impedance matching at higher frequency. In this case, lower frequency band is considered for liquid sensing applications. Moreover, Fig. 17b shows that the absorption peak shifts linearly from the higher to a lower frequency with increasing permittivity values. Comparing with existing works it can be said that the proposed absorber can be applied for other applications like agriculture, industry, radar, and satellite sensors.

So, the proposed SSRR with microstrip line MMA compared with various recent works is shown in Table 2, where MMA patch design, unit cell size, thickness, operation frequency band, absorption, and polarization insensitivity are noted. Moreover, most of the existing MMA works at different frequency bands without any specific application, where only^15,36^ is for dedicated Ku-band application but suffers from lower polarization insensitivity and larger unit cell size. The proposed MMA unit cell is found comparatively smaller in size than^15,36,39,41^ and realizes two absorption frequency bands in the Ku spectrum region, where 99.99% absorption was realized at the Ku band with 90° polarization insensitivity, which is a good performance of the proposed MMA compared with others in Table 2. Detail’s analysis of metamaterial property of proposed design is investigated and found DNG value for both frequency bands. This excellent absorption property of the proposed MMA makes it a better candidate for the absorption and sensing application of Ku-band.

**Table 2:** Comparison of proposed MMA with existing works.

References	Patch type	Substrate material	Unit cell size	Operating frequency	Resonant frequency	Maximum absorption	Polarization angle insensitivity	Applications
^4^	Square spiral shape	FR-4 4.4	0.14λ × 0.14λ × 0.0608λ	Ku K	12.2 17.1 19.7	99.8	θ ≤ 50°	Absorber
^35^	T shape	FR-4 4.4	0.4λ × 0.4λ × 0.027λ	X Ku	10.19	99.9%	θ ≤ 60°	Absorber
	SI shape		0.36λ × 0.36λ × 0.024λ		9.32 10.75			
	Split Jerusalem Cross		0.36λ × 0.36λ × 0.024λ		–			
^36^	Shorted stub CR MMA	FR-4 4.6	0.50λ × 0.50λ × 0.023λ	Ku	17 18	99.9% 99.83	θ ≤ 70°	Absorber
^37^	CSR and CCSR	FR-4 4.35	0.35λ × 0.35λ × 0.0226λ	X Ku	11.15 16.1	99.9%	θ ≤ 60°	Absorber
^38^	CRR	FF-4 4.2	0.19λ × 0.19λ × 0.052λ	X Ku	8.12 12.39	99.66%	θ ≤ 15°	Absorber
^39^	Jerusalem cross	FR-4 4.4	1.18λ × 1.18λ × 0.079λ	Ku	14.75 15.1 16.25	95%	θ ≤ 60°	Absorber
^4^°	Eight-Resistive arm cell	FR-4 3.9	0.41λ × 0.41λ × 0.082λ	X Ku	9 11 13	99%	θ ≤ 65°	Absorber
^41^	Jerusalem cross with SRR	FR-4 4.4	0.69λ × 0.69λ × 0.046λ	X Ku	8.6 10.2 11.95	99.98%	θ ≤ 60°	Absorber
^29^	Concentric closed CRR	FR-4 4.4	0.185λ × 0.185λλ0.01λ	C X Ku	5.57 7.96 13.44	99.28%	θ ≤ 75°	Absorber
^31^	Four-fold resonator	FR-4 4.3	0.37λ × 0.37λ × 0.059λ	X Ku	11.31 14.23 17.79 17.81	99.15%	θ ≤ 90°	Absorber
^15^	Ring C shape quasi-MMA	FR-4 4.6	0.92λ × 0.92λ × 0.072λ	Ku	13.78 15.3	99.6%	θ ≤ 60°	Absorber and sensing
Proposed	SSRR with micro strip line	FR-4 4.3	0.44λ × 0.44λ × 0.088λ	Ku	14.62 16.30	$99.99$%	θ ≤ 90°	Absorber and sensing

## Conclusions

This article proposed an SSRR metamaterial absorber for a dedicated Ku-band application. The unit cell size is 0.44λ × 0.44λ × 0.088λ, which is smaller than existing dedicated Ku-band MMAs. Two perfect absorption fractional bandwidths of 2.87% and 3.06% are achieved at 14.42–14.84 GHz and 16.06–16.54 GHz frequency bands, respectively. DNG metamaterial properties are also achieved for each absorption frequency band with wide polarization insensitivity. The PCR value of the proposed MMA shows that the proposed MMA is absolutely an absorber rather than a polarization converter, and interference theory was calculated to verify the absorption behavior. An increased EM wavefield with distributed surface charge is also attained by the proposed structure, which makes excellent plasmonic coupling capacitance and inductance between the patch and ground plane. Moreover, permittivity sensor application of proposed MMA is investigated, and a good response was found with changing the dielectric constant of material under test. Therefore, the proposed MMA can be an ideal candidate for the relevant radio and satellite communication applications, radar imagining, frequency identification, higher frequency noise reduction, signal detection, sensor applications, etc.
